# Spatial Neglect Midline Diagnostics From Virtual Reality and Eye Tracking in a Free-Viewing Environment

**DOI:** 10.3389/fpsyg.2021.742445

**Published:** 2021-11-29

**Authors:** Bastian I. Hougaard, Hendrik Knoche, Jim Jensen, Lars Evald

**Affiliations:** ^1^Department of Architecture and Media Technology, Aalborg University, Aalborg, Denmark; ^2^Neurocenter Østerskoven, Hobro, Denmark; ^3^Hammel Neurorehabilitation Centre and University Research Clinic, Hammel, Denmark

**Keywords:** hemispatial neglect, virtual reality immersion therapy, diagnostic techniques and procedures, unilateral spatial neglect, eye tracking, head rotation, stroke, acquired brain injury

## Abstract

**Purpose:** Virtual reality (VR) and eye tracking may provide detailed insights into spatial cognition. We hypothesized that virtual reality and eye tracking may be used to assess sub-types of spatial neglect in stroke patients not readily available from conventional assessments.

**Method:** Eighteen stroke patients with spatial neglect and 16 age and gender matched healthy subjects wearing VR headsets were asked to look around freely in a symmetric 3D museum scene with three pictures. Asymmetry of performance was analyzed to reveal group-level differences and possible neglect sub-types on an individual level.

**Results:** Four out of six VR and eye tracking measures revealed significant differences between patients and controls in this free-viewing task. Gaze-asymmetry between-pictures (including fixation time and count) and head orientation were most sensitive to spatial neglect behavior on a group level analysis. Gaze-asymmetry and head orientation each identified 10 out of 18 (56%), compared to 12 out of 18 (67%) for the best conventional test. Two neglect patients without deviant performance on conventional measures were captured by the VR and eyetracking measures. On the individual level, five stroke patients revealed deviant gaze-asymmetry within-pictures and six patients revealed deviant eye orientation in either direction that were not captured by the group-level analysis.

**Conclusion:** This study is a first step in using VR in combination with eye tracking measures as individual differential neglect subtype diagnostics. This may pave the way for more sensitive and elaborate sub-type diagnostics of spatial neglect that may respond differently to various treatment approaches.

## 1. Introduction

Globally, there is an annual incidence of about 16.9 million first-ever strokes and 33 million stroke survivors (Feigin et al., 2014). Stroke is a leading cause of cognitive impairments as approximately one third of stroke survivors live with life-long disability (Singh et al., 2018). Spatial neglect represents a common impairment following stroke affecting at least 30% of stroke survivors (Hammerbeck et al., 2019). However, SN often goes under-diagnosed and consequently under-treated (Bowen et al., 1999; Edwards et al., 2006; Chen et al., 2013). Spatial neglect (SN) constitutes a heterogeneous syndrome with several different, dissociable symptoms or subtypes (Buxbaum et al., 2004; Kerkhoff and Schenk, 2012; Rode et al., 2017). Conventional tests often simply assess one aspect of these underlying deficits. No single conventional neglect test can reliably diagnose all patients, i.e., one patient may pass the first four tests and fail the fifth, another may fail the first and pass the rest. This often relates to different subtypes of neglect e.g., motor and sensory neglect or ego- and allocentric (body and object centered) neglect. Egocentric neglect manifests itself as inattention to stimuli presented in the contralesional hemispace of different body midlines (trunk, head and eyes) and allocentric neglect as inattention to the contralesional half part of objects regardless of their egocentric placement. Many of these subtypes differ in diagnostic measures and prognostic consequences. Ego- and allocentric neglect seem to have different recovery rates (Demeyere and Gillebert, 2019) and different neglect midlines may require different treatment approaches, e.g., oculomotor neglect.

Virtual Reality (VR) combined with eye tracking may be a useful technology to detect different aspects of SN in complex 3D environments. The ability to control and monitor all motor and sensory input and output in high spatial resolution and temporal millisecond precision level may be ideal for assessing subtle impairments in spatial attention. VR has for long been a target for recording diagnostics of cognitive impairments in cognitive neuroscience, although pen-and-paper tests still dominate clinical neuropsychology. Digital tests can provide novel measures to quantify neglect that are too cumbersome to compute from pen and paper tests in clinical contexts.

A literature review by Negut et al. (2016) confirmed virtual reality to be a sensitive neuropsychological assessment tool in detecting cognitive impairment for clinical practice. They identified *task performance indicators* based on: 1) time measured, 2) number of errors in performing a task, 3) quantitzation of head or body movement. Only one study used head movement, the rest used task-based parameters. A number of studies has explored VR measures to assess neglect, through task based measures, either for training cognitive functions or making assessments (Nolin et al., 2019). For example, Broeren et al. (2007) used cancellation tasks to derive the *pattern of search* and Yasuda et al. (2020) used object detection tasks to assess near- and far SN. To assess neglect, eye tracking can be used in conjunction with head-mounted displays to scan eye movement patterns (Baheux et al., 2004, 2006). Kim et al. (2004) combined eye tracking with VR task to create a diagnostics tool for SN. Twelve patients and 40 controls were diagnosed based on deviation angle (between mandated and actual gaze position), no-attention time, scanning time, number of cues, failure rate of mission, and ratio of right/left scan. Their deviation angle correlated with line bisection test results. Other studies have used eye tracking in non-VR environments to assess neglect, for example Cazzoli et al. (2016) who measured x-axis gaze position while participants with neglect and visual field defect (VFD) viewed a projected virtual traffic scene. The neglect patients with VFD showed a significant rightward deviation in x-axis gaze position compared to those with no symptoms. But their study did not include neglect patient without visual field defects and all participants used chin rests removing the possibility of head movements. Ptak et al. (2009) assessed neglect with free-viewing of photographs, but used a chin-rest and found a group-level ipsilesional shift in the fixation distribution for the patient group. Results from eye tracking have been promising but not without contradictions. For example, Primativo et al. (2015) found no differences in the number and durations of fixations between SN and non-SN patients in a free viewing task of an albeit asymmetric picture. Studies use different measures derived from gaze measurements to identify neglect, such as re-fixations, mean amplitude and saccade landing position (Paladini et al., 2019) and horizontal fixation frequency (Ptak et al., 2009).

Contrary to setups in previous studies, this study provides both head-mounted immersive VR and eye-tracking that allows for unconstraint head and eye movement. We wanted to investigate to what extent SN can be assessed based on basic continuous measurements of head and eye movement while patients are spontaneously and freely looking around in a simple immersive VR environment and whether these measures can detect individual motor biases across different body midlines.

We hypothesized that virtual reality and eye tracking may be used to assess sub-types of spatial neglect in stroke patients not readily available from conventional assessments. Specifically, the aim was to investigate whether differences in attentional biases across different frames of references (egocentric midlines of body, head, eyes, and allocentric midlines of objects) can be assessed by VR and eye tracking on a group level (patients and controls) and individual level (subtypes diagnostics).

## 2. Methods

All participants were recruited at Hammel Neurorehabilitation Centre (HNC) and University Research Clinic. Patients with right hemisphere brain injury and behavioral symptoms of SN (with KF-NAP scores larger than zero) were included. KF-NAP was used as a baseline measure to identify neglect patients, due to its high sensitivity to neglect symptoms direct relation to everyday activities and changes in the severity of neglect symptoms during recovery from stroke patients (Chen et al., 2015). Patients with previous brain injury or neurodegenerative diseases as well as bedridden and blind patients were excluded. Healthy age and gender matched controls were recruited from the staff at HNC.

### 2.1. Conventional Measures

Conventional SN tests were applied for comparison to virtual reality measurements. The **Line bisection test** from the Behavioral Inattention Test (Wilson et al., 1987) requires the patients to mark the center of each of three (8 inches 20.3 mm) horizontal lines that are printed on a sheet of white paper. In the **Apples Cancellation Test** (Bickerton et al., 2011) patients are instructed to cancel out targets depicting outlines of apples, but only complete apples without gaps, on a sheet of white paper. The test yields individual scores for both non-lateralized visual attention (accuracy), egocentric and allocentric neglect. The **gray scales gradient test** requires patients to judge which of two left-right mirror-reversed gray scale gradients appears darker. For each of the 12 pairs of stimuli, one of the grayscales is shaded from white on the left to black on right, and the other is shaded in the opposite direction. The task is highly sensitive to unilateral hemispheric brain damage, and can uncover attentional biases in patients without SN symptoms on conventional cancellation or line bisection tests (Mattingley et al., 2004). The **chimeric faces test** consists of 12 pairs of chimeric faces generated from portraits of 10 different people smiling and 10 portraits of the same people with a neutral expression arranged vertically (Mattingley et al., 1993; Sarri et al., 2006). Each pair contains two chimeras of the same person, one neutral in the left half and smiling in the right half, and the other vice versa, with the vertical position counterbalanced. Patients are instructed to chose the happier of the two thus revealing left (20/0) or right (0/20) attentional biases. The **KF-NAP** was developed on the basis of the Catherine Bergego Scale (Azouvi, 1996)—the most widely used behavioral assessment instrument for SN (Azouvi, 2017). KF-NAP is a manual method for systematic observation of SN during everyday activities, including 10 categories: gaze orientation, limb awareness, auditory attention, dressing, grooming, personal belongings, navigation, collisions, having a meal, and cleaning after meals (Chen et al., 2012, 2015). Each category is scored from 0 to 3, with higher scores indicating more severe neglect. The sum score ranging from 0 to 30 indicates the severity of SN with predefined cut-off scores of the severity (0 = none, 1–10 = mild, 11–20 moderate, 21–30 severe). KF-NAP has been shown to be very sensitive to neglect symptoms directly related to everyday activities and to changes in the severity of neglect symptoms as stroke patients are recovering (Chen et al., 2015).

### 2.2. Virtual Reality and Eye Tracking Procedure and Apparatus

**Figure 1:**
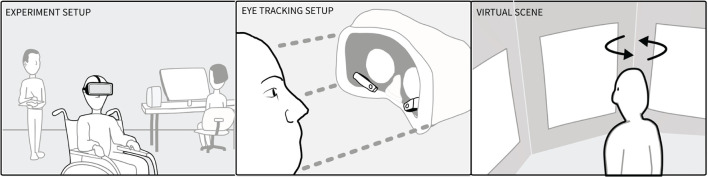
**(Left)** Experimental setup showing note taker, technician and the participant who wore a head-mounted display whilst sitting in a wheel chair. **(Middle)** Participants wore an HTC Vive with Pupil Labs eye tracking cameras mounted inside. **(Right)** The virtual museum contained the participant and three images to the left (−48°), right (+48°), and middle (0°).

Participants were instructed about wearing a virtual reality headset and the free viewing task: “In a moment you will be placed in a museum. You get a few minutes to look around. You do not have to describe what you see.” We then equipped the participants with the virtual reality headset (Figure 1, Left) and started an eye tracking calibration procedure (staring at dots shown in the head-mounted display). After successful Pupil Labs 2D calibration, they were presented with a simple museum environment (Figure 1, Right). The environment consisted of three sets of three pictures shown on three walls. Head movement was needed to see the entirety of the left and right pictures. The picture sets were either three faces of well known persons, three similar seasonal colored landscapes, or three non-figurative paintings. The picture sets were selected to reflect symmetry of salience, were placed equally far from the middle and counterbalanced. The participants viewed each picture set for 60 s, totalling 180 s of free viewing time. While participants looked around, we logged the VR headset's position and orientation and their gaze. We used an HTC Vive VR headset with Pupil Labs eye tracking equipment, which tracks with 1 degree accuracy (Pupil Labs, 2021). The virtual environment was developed in Unity, which recorded the sensor data from the VR headset and raw raycasted gaze data using Pupil Labs' API without jitter postprocessing. The visual angle of each picture subtended 32° in width and 40° in height. The pictures were spaced 16° apart. The HTC Vive provided 110° field of view, although this depended on the fit (Lynn et al., 2020). Data from VR and Eye trackers were recorded at a 30 Hz sampling rate and analyzed with the saccades package in R.

### 2.3. Virtual Reality and Eye Tracking Measure Description, Preparation, and Analysis

After their calculation, all measures were normalized to range from −1 (leftward) to 1 (rightward). For all measures except the fixations, we subtracted the percentage of time spent on the left side from the percentage of time spent on the right side (see Table 1). For these measures we defined impairments through cut-off criteria based on the difference between the percentages of time spent in the left and the right hemispace in the control group (e.g., including the 5th percentile or none of the controls). These support clinical diagnostics e.g., whether patients had allo-, egocentric neglect, or neglect related to head or eye midline deviations.

**Table 1:** Virtual reality and eye tracking measurements.

**VR measure**	**Description**	**Interpretation**	
Gaze asymmetry between-pictures	Estimated gaze from eye and head while comparing looking at the left and right picture.	Egocentric-neglect (body midline)	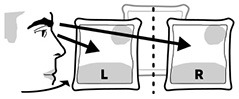
Gaze asymmetry within-picture	Difference in time spent on estimated gaze from eye and head combined while looking at the pictures.	Allocentric neglect (object midline)	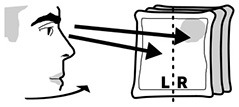
Head orientation left/right	Amount of time spent with the head rotated to the left/right.	Caputomotor neglect (head midline)	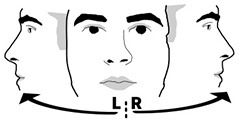
Eye orientation left/right	Hemispheric orientation of the eyes only, without considering fixations or gaze in scene.	Oculomotor neglect (eye midline)	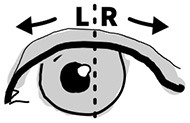
Fixation duration left/right	Duration of eye fixations within the scene. Analyzed from saccades (λ* =* 1).	Egocentric neglect (body midline)	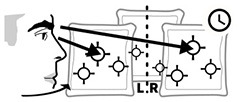
Fixation count left/right	Number of eye fixations made left/right within the scene.	Egocentric neglect (body midline)	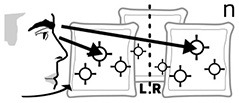

Eye tracking data was filtered to include only data points from looking at the three pictures. Gaze asymmetry measured the position of the participants' gaze projected onto these pictures. *Between picture gaze asymmetry* left out eye tracking data from the middle picture and subtracted the amount of time spent looking at the left-most picture from the time looking at the right-most picture. These temporal aggregates were solely based on the number of eye tracking samples located on each respective picture. Their fixation counts and the totalled duration of the fixations were separate measures. *Within-picture gaze asymmetry* divided each picture into a left and right section and compared the participants' time spent gazing on each side, to measure allocentric neglect.

Head- and eye orientation were measured irrespective of gaze in virtual reality in order to assess motor neglect related to different body midlines. We did not correct for head position when using head orientation as the patients were seated in wheel chairs and potentially not sitting fully upright. *Head Orientation L/R* subtracted the percentage of time (in seconds) participants spent with the head rotated to the right from the percentage of time spent on the left. For example, if a participant's head was oriented to the right side twice as long (66%) as to the left side (33%), this measure reported an imbalance of −33%. *Eye Orientation L/R* was based on how much time the person was looking to the left of their visual field center line (where the nose is pointing) in comparison to looking right of it aggregated over all three pictures independent of head rotation. Fixation durations and counts are common aggregate measures of gaze data but can behave differently. One could spend equal amounts of gaze time left and right, yet still have fewer fixations and longer durations of fixations to one side. Subtracting the percentage of fixations that happened on the left from those on the right providing the measures *fixation count L/R*. The measure *fixation duration L/R* relied on the difference of the sums of fixation durations from the left and the right divided by the sum of all fixation durations.

### 2.4. Statistical Approach

The conventional tests produced continuous raw scores for the Apples Cancellation and the Line bisection tests, and asymmetry scores for the gray scale gradients and the Chimeric faces. They were analyzed according to their normative cut-off scores from the literature and binary neglect diagnostics were calculated. For the VR and eye tracking measures we relied on the data from the control group. For each measure we derived cut-offs (*cu*) by adding the mean *m*_*c*_ of the control group to 1.645 times its standard deviation *sd*_*c*_ (95th percentile): *cu* = *m*_*c*_±1.645*sd_*c*_. Mann-Whitney tests checked for between group differences unless indicated otherwise. An alpha value <5% (*p* < 0.05) was considered statistically significant. Two-sided testing with a 5% alpha value (α = 0.05) and 80% power (1−β = 0.80) along with enrolling 18 patients and 16 healthy controls allowed us to detect large effect sizes (Cohen's d > 1.0).

### 2.5. Participant Characteristics

Twenty three stroke patients and 16 age and gender matched controls from a hospital staff population were recruited. However, five patients were excluded for different reasons: one had a premorbid surgical fixation of the neck, that prevented natural movement of the head; one was easily fatigued and the VR procedure was aborted; one got emotional labile during testing with the conventional test and VR testing was not performed; one patient could not be satisfyingly calibrated for the eye tracking; and one patient had left-sided brain damage and right-sided neglect. Participants were matched to have equal age and gender in the patient (range 51–74, *M* = 61.4, *SD* = 6.6, 9F/9M) and control group (range 52–69, *M* = 60.0, *SD* = 4.8, 8F/8M), with no significant difference for age according to a *t*-test (*p* = 0.491). The patient demographic characteristics are presented in Table 2. Written informed consent was obtained from the participants.

**Table 2:** Patient demographics and brain injury characteristics.

**ID**	**Sex**	**Hand**	**Age**	**Days since injury**	**Lesion**
2	M	R	56	173	Traumatic subarachnoid hemorrhage and right subdural hematoma
4	F	R	66	37	Hemorrhage right basal ganglia
7	M	R	58	62	Subarachnoid hemorrhage
8	M	R	56	55	Infarction right occipital lobe
16	M	R	63	15	Infarction right internal carotid artery and middle cerebral artery
17	F	R	61	74	Large hemorrhage right hemisphere
21	F	L	54	52	Hemorrhage right hemisphere frontal
24	F	R	68	25	Infarction right hemisphere frontal
26	M	R	56	30	Infarction right middle cerebral artery
28	F	R	69	31	Hemorrhage right thalamus
32	F	R	74	497	Infarction right hemisphere
33	F	R	58	100	Large hemorrhage right basal ganglia
34	F	R	73	23	Infarction right middle cerebral artery
35	F	R	63	100	Large infarction right frontal and parietal lobe
36	F	R	58	54	Infarction right basal ganglia and parietal lobe, thrombus right internal carotid artery and middle cerebral artery
37	M	R	51	154	Infarction right middle cerebral artery
38	M	R	57	61	Infarction right middle cerebral artery
39	M	R	64	17	Hemorrhage right middle and frontal

## 3. Results

### 3.1. Group-Level Results

**Figure 2:**
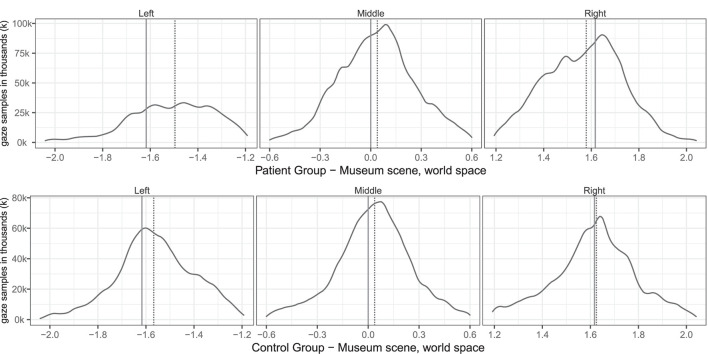
Group level spatial distribution of gaze samples on the (solid line) and median gaze (dotted line) of patients and controls. The *x*-axis spans the horizontal world space of the virtual museum scene from −2.0 to 2.0. The *y*-axis plots the corresponding gaze sample counts in thousands (k).

Table 3 describes the means and *p*-values from Mann-Whitney tests on both conventional and VR measures used in the study. All conventional measures were significantly different (*p* < 0.05) between the patient and control groups. Most virtual reality measures differed significantly, too, except gaze asymmetry within picture and eye orientation left/right. The correlations between virtual reality measures are described in Table 4. Figure 2 depicts the density plots of the three pictures split by patient and control groups.

**Table 3:** Group-level reports for patient and control groups (conventional and VR tests).

	**Patients (18)**		**Controls (16)**			
**Measurement**	**N/Mean**	**SD**	**N/Mean**	**SD**	**Cut-off scores**	***p*** **-value**
KF-NAP	13.6	5.8	–	–	–	–
Line bisection	6.3	3.1	8.9	0.3	≤ 7	0.001^*^
AC accuracy	32.0	14.0	48.5	2.0	≤ 41	0.000^*^
AC asymmetry egocentric	7.4	6.6	0.1	1.5	≥±3	0.001^*^
AC asymmetry allocentric	3.1	4.9	0.0	0.0	≥±2	0.006^*^
Gray scales (average left/right)	4/16	–	9/11	–	≥1/19	0.002^*^
Chimeric faces (average left/right	7/13	–	12/8	–	≥1/19	0.020^*^
Gaze asym. between-pict.	0.22	0.25	0.01	0.05	−0.07 / 0.09	0.003^*^
Gaze asym. within-pict.	0.09	0.41	0.14	0.26	−0.28 / 0.57	0.665
Head Orientation L/R	0.21	0.23	0.01	0.05	−0.07 / 0.10	0.002^*^
Eye orientation L/R	0.15	0.29	0.08	0.20	−0.25 / 0.40	0.391
Fixation duration L/R	0.30	0.33	0.08	0.22	−0.29 / 0.44	0.028^*^
Fixation count L/R	0.30	0.30	0.08	0.18	−0.21 / 0.37	0.012^*^

*Cut-off scores for conventional test based on normative data. Fifth and 95th percentiles for VR and ET measures are based on control group performances.*

* *indicates significance (p < 0.05)*.

**Table 4:** Correlations between VR measures, calculated using a pearson correlation coefficient.

	**Gaze asymmetry within pictures**	**Head orientation, duration L/R**	**Eyes, duration L/R**	**Eye Fixations, duration L/R**	**Eye fixations, count L/R**
Gaze asymmetry between pictures	–0.39	1.00	0.11	0.68	0.77
Gaze asymmetry within pictures	–	–0.35	0.50	0.22	0.20
Head orientation, duration L/R		–	0.11	0.69	0.78
Eyes, duration L/R			–	0.49	0.49
Eye fixations, duration L/R				–	0.92
Eye fixations, count L/R					–

### 3.2. Individual Results

**Figure 3:**
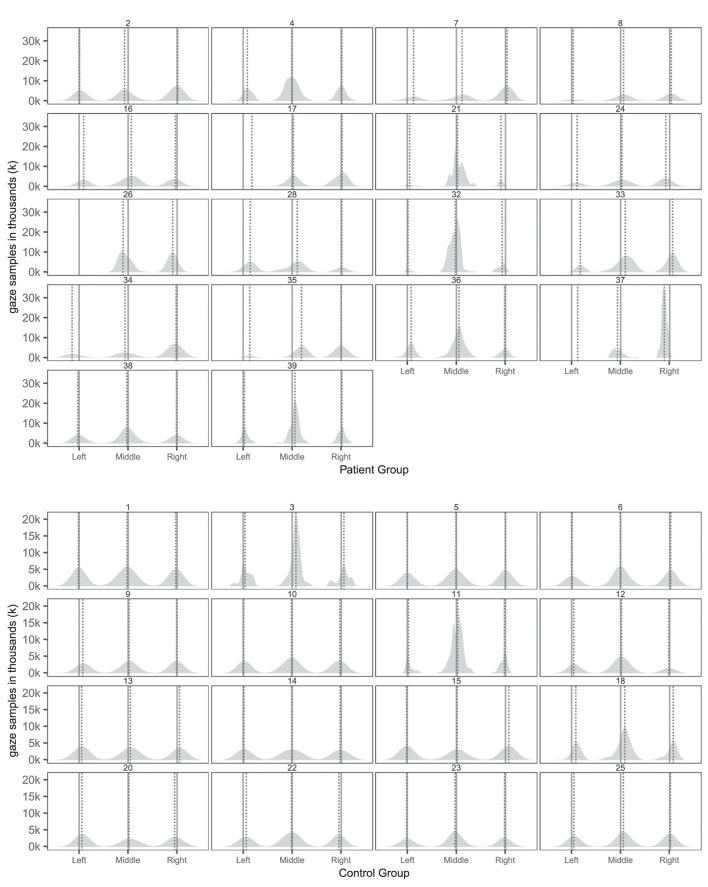
Individual spatial distributions of gaze samples on the three pictures with true picture midline (solid lines) and median gaze (dotted lines) of patients and controls. The *x*-axis reports the horizontal axis of the virtual museum scene. The *y*-axis counts the number of gaze samples in thousands (k).

Tables 5, 6 provides an overview of how well the conventional and virtual reality tests identified individual attentional biases in the patient and control groups. Figure 3 show individual gaze distributions.

**Table 5:** Patient group demographics and results from conventional and VR measures.

** ID (18 Patients)**	**2**	**4**	**7**	**8**	**16**	**17**	**21**	**24**	**26**	**28**	**32**	**33**	**34**	**35**	**36**	**37**	**38**	**39**
KF-NAP sum score	6	16	18	15	18	16	4	20	16	9	16	16	13	15	4	26	8	9
- gaze orientation	1	1	2	2	2	2	1	2	2	1	2	1	1	2	0	3	1	0
- limb awareness	1	2	2	1	3	2	1	2	2	2	2	2	2	1	1	3	1	2
- auditory attention	0	1	1	2	2	1	0	2	1	0	1	1	1	1	0	2	0	0
- personal belongings	1	2	2	2	2	2	0	2	2	1	2	2	1	2	1	3	1	0
- dressing	0	1	2	1	2	2	0	2	2	0	2	2	2	2	1	3	2	2
- grooming	1	1	2	1	1	1	1	2	2	1	1	2	2	2	0	2	1	2
- navigation	1	2	2	2	-	2	0	3	2	2	-	2	2	1	0	3	2	-
- collisions	1	2	3	2	1	2	0	2	2	2	1	2	2	1	1	3	0	-
- meals	0	2	1	1	1	1	0	1	1	0	2	1	0	1	0	2	0	0
- cleaning after meals	0	2	1	1	2	1	1	2	0	0	1	1	0	2	0	2	0	-
Line bisection	6	9	9	0	5	0	4	6	4	9	3	9	9	8	8	7	9	9
AC accuracy	13	35	36	10	38	20	47	18	7	48	18	37	42	42	48	29	41	47
AC asymmetry egoc.	13	15	14	10	11	14	3	4	7	2	14	13	–3	–3	0	16	2	1
AC asymmetry alloc.	0	1	16	0	3	3	0	2	3	0	9	0	–1	8	0	12	0	0
Gray scales left/right	18/2	2/18	0/20	0/20	0/20	20/0	0/20	0/20	0/20	0/20	0/20	0/20	11/9	0/20	0/20	1/19	4/16	12/8
Chimeric faces L/R	11/9	7/13	0/20	3/17	4/16	20/0	1/19	10/10	8/12	9/11	10/10	0/20	16/4	4/16	0/19	14/6	6/14	11/9
Gaze Asym. Between-Pict.	0.14	0.04	0.35	0.35	0.05	0.52	0.04	0.23	0.44	–0.19	0.08	0.27	0.43	0.42	–0.08	0.79	–0.01	0.03
Gaze Asym. Within-Pict.	–0.09	0.16	0.45	0.37	0.20	0.20	0.08	–0.08	–0.61	0.61	–0.18	0.61	–0.28	0.47	0.37	–0.84	–0.25	0.47
Head Orientation L/R	0.14	0.05	0.36	0.35	0.05	0.52	0.03	0.23	0.43	–0.17	0.09	0.30	0.43	0.39	–0.07	0.67	–0.01	0.04
Eye Orientation L/R	0.06	0.09	0.29	0.43	0.28	–0.06	0.21	0.21	–0.28	0.40	–0.36	0.62	–0.21	0.09	0.10	0.48	–0.23	0.57
Fixations, time L/R	–0.07	–0.10	0.63	0.58	0.25	0.48	0.20	0.11	0.20	–0.07	–0.09	0.59	0.62	0.95	0.14	0.70	–0.17	0.42
Fixations, Count L/R	0.06	0.02	0.51	0.52	0.22	0.67	0.13	0.41	0.12	–0.09	–0.04	0.54	0.51	0.86	0.17	0.67	–0.19	0.33

*Underlined numbers are significantly different (beyond the normative threshold from the controls). Blue shading denotes bias to the right. Red shading denotes bias to the left*.

**Table 6:** Control group demographics and results from conventional and VR measures.

**ID (16 Controls)**	**1**	**3**	**5**	**6**	**9**	**10**	**11**	**12**	**13**	**14**	**15**	**18**	**20**	**22**	**23**	**25**
Line bisection	8	9	9	9	9	9	9	9	9	9	9	9	9	9	9	9
AC accuracy	50	45	48	50	50	49	47	50	50	43	50	50	49	48	48	49
AC asymmetry egoc.	0	3	0	0	0	0	3	0	0	-2	0	0	1	-2	-2	1
AC asymmetry alloc.	0	0	0	0	0	0	0	0	0	0	0	0	0	0	0	0
Gray scales L/R	8/12	16/4	12/8	17/3	6/14	16/4	6/14	5/15	8/12	7/13	19/1	1/19	5/15	12/8	5/15	5/15
Chimeric faces L/R	10/10	13/7	11/9	15/5	17/3	11/9	5/15	17/3	6/14	10/10	18/2	18/2	14/6	11/9	10/10	2/18
Gaze Asym. Between-Pict.	–0.01	0.00	0.04	0.10	0.06	0.04	0.06	–0.11	–0.05	0.00	0.01	0.01	–0.05	0.03	0.01	0.05
Gaze Asym. Within-Pict.	–0.19	0.53	–0.07	0.14	0.22	–0.10	0.11	0.14	0.41	–0.02	0.19	0.66	0.14	–0.06	–0.23	0.42
Head Orientation L/R	–0.02	0.01	0.05	0.09	0.07	0.05	0.08	–0.11	–0.04	0.00	0.02	–0.01	–0.05	0.02	0.00	0.06
Eye Orientation L/R	–0.21	0.43	0.14	0.14	0.16	0.01	0.25	–0.09	–0.19	–0.11	–0.01	0.45	–0.07	0.01	0.07	0.24
Fixations, Time L/R	0.00	0.46	–0.06	0.05	0.12	0.14	0.27	0.16	0.08	–0.26	–0.22	0.36	–0.25	0.01	–0.02	0.39
Fixations, Count L/R	–0.07	0.33	–0.03	0.12	0.23	0.02	0.35	–0.10	0.01	–0.03	0.03	0.32	–0.15	–0.05	–0.05	0.35

*Numbers underlined are above the 95th or below the 5th percentile (false positives). Blue shading denotes bias to the right. Red shading denotes bias to the left*.

### 3.3. Gaze Asymmetry

**Figure 4:**
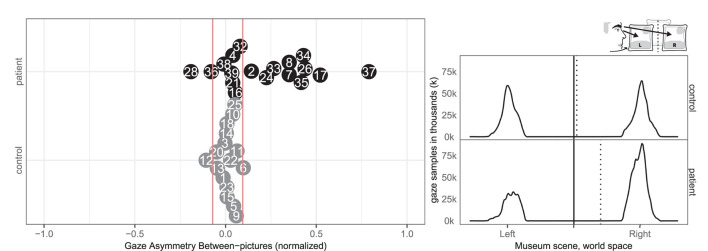
Gaze Asymmetry Between-pictures. **(Left)** Individual normalized time difference between looking at left/right picture in patient (black, top) and control (gray, bottom) with normative threshold cut-offs (red lines). **(Right)** Patient (black, top) and control (gray, bottom) histograms of horizontal gaze sample positions limited to the left and right painting with the true (solid) midline and the (dotted) group median of horizontal gaze positions.

Ten patients (2, 7, 8, 17, 24, 26, 33, 34, 35, 37) and one control (6) had abnormal right-ward bias related to the viewing time of the left and right most pictures, whereas two patients (28, 36) and one control (12) had small left-ward biases contrary to expectations (Figure 4). Right-ward biases could be interpreted as SN behaviors centered at the body midline (egocentric neglect).

**Figure 5:**
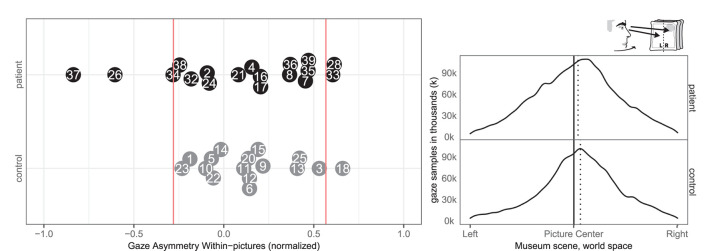
Gaze Asymmetry Within-picture. **(Left)** Individual time spent looking right and left within all pictures in patient (black, top) and control (gray, bottom) with the empirical cut-offs (red lines). **(Right)** Spatial distribution of gaze samples within all pictures with true (solid) midline and group median (dotted).

Two patients (28, 33) and none of the controls had right-ward bias related to the viewing time within the left and right half part of the pictures, whereas three patients (26, 34, 37) and one control (18) had left-ward bias (see Figure 5). Even though patients more commonly had deviant within-picture bias than controls, the biases were both right-ward and left-ward and two patients (37, 26) had fairly large left-ward biases contrary to expectation. This measure could be interpreted as SN behaviors related to the object midline (allocentric neglect), even though the behavior of some patients was contrary to expectation.

### 3.4. Head- and Eye-Orientation

**Figure 6:**
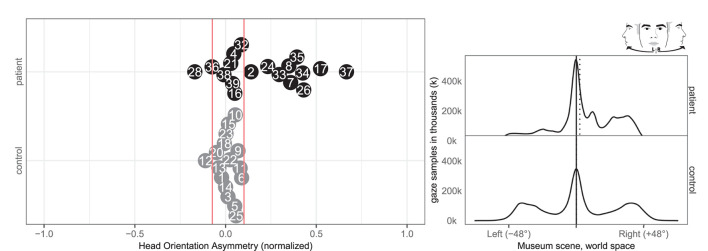
Head orientation left/right. **(Left)** Individual time spent rotating head left and right in patient (black, top) and control (gray, bottom) with normative threshold cut-offs (red lines). **(Right)** Spatial distribution of patient and control head orientation with true (solid) midline and group median (dotted).

In terms of head orientation, 11 of the patients (2, 7, 8, 17, 24, 26, 33, 34, 35, 36, 37) and none of the controls showed an abnormal right-ward bias, whereas one patient (28) and one control (12) had a small abnormal left-ward bias (see Figure 6). Right-ward bias of head orientation can be interpreted as motor neglect or lack of intention to initiate head movements toward the left.

**Figure 7:**
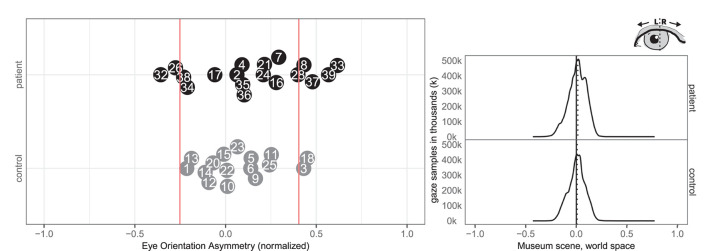
Eye orientation left/right. **(Left)** Individual time spent rotating eye balls left and right in patient (black, top) and control (gray, bottom) with normative threshold cut-offs (red lines). **(Right)** Spatial distribution of patient and control eye ball orientation with true (solid) midline and group median (dotted).

Four patients (8, 33, 37, 39) and two controls (3, 18) had an abnormal right-ward eye position bias, i.e., the eyes spend more time looking to the right than the left regardless of head movement whereas two patients (26, 32) and none of the controls had left-ward eye movement biases (see Figure 7). Right-ward eye movement bias could be interpreted as oculomotor neglect or a lack of intention to move ones eyes to the left.

### 3.5. Fixation-Duration and Fixation-Count

**Figure 8:**
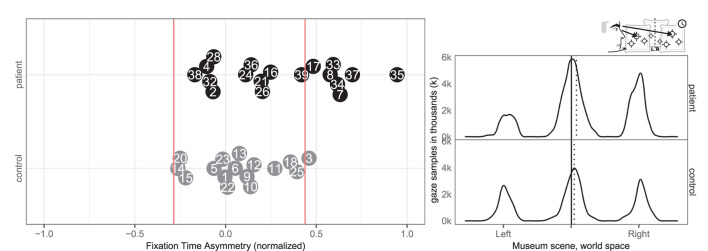
Fixation left/right. **(Left)** Individual time spent fixating eyes in the left and right for patient (black, top) and control (gray, bottom) with normative threshold cut-offs (red lines). For example, –1 indicates that participants spent 100% of the time fixating on the left. **(Right)** Time-weighted spatial distribution of patient and control fixations, according to world midline, with time-weighted medians (dotted).

Seven patients (7, 8, 17, 33, 34, 35, 37) and one control (3) had abnormal right-ward fixation time bias, i.e., they spent more time on each fixation in the right VR hemispace compared to the left, whereas no patients and no controls exhibited left-ward biases (see Figure 8).

**Figure 9:**
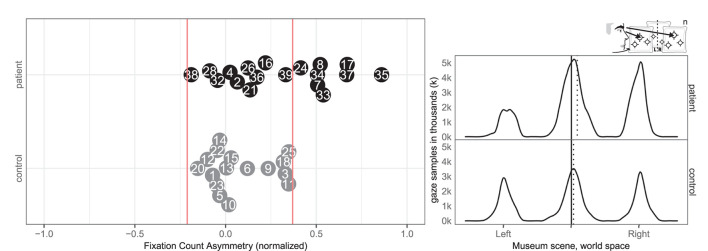
Fixation count left/right. **(Left)** Individual fixation count right/left for patient (black, top) and control (gray, bottom) with normative threshold cut-offs (red lines). **(Right)** Spatial distribution of patient and control fixation counts, according to the world (solid) midline with (dotted) median.

Likewise, eight patients (7, 8, 17, 24, 33, 34, 35, and 37) and none of the controls had abnormal right-ward fixation counts, interpreted from the number of saccades, whereas no patients and no controls had left-ward bias (see Figure 9).

## 4. Discussion

This study aimed at investigating whether attentional biases across different egocentric midlines of body, head, eyes, and allocentric midlines of objects could be assessed by VR and eye tracking on a group and individual level in patients with SN.

On a group level, gaze asymmetry between-pictures (egocentric neglect) was highly sensitive but gaze asymmetry within-pictures (allocentric neglect) was not. Likewise, head orientation (caputomotor neglect) was highly sensitive, though eye orientation (oculomotor neglect) was not. Finally, fixation time and fixation count were both highly sensitive to right-ward neglect behavior.

On an individual level, even though gaze asymmetry within-pictures (allocentric neglect) was not a significant measure of neglect behavior on a group level, five patients as opposed to one control did in fact revealed deviant behavior in either direction. Likewise did six patients as opposed to two controls revealed deviant eye orientation behavior in either direction.

Overall, gaze-asymmetry between-pictures and head orientation each identified 10 out of 18 (56%) of the stroke patients, compared to 12 out of 18 (67%) for the best of the conventional tests. Interestingly, three patients (34, 38, and 39) were not picked up by any of the conventional neglect test at all, apart from their low to moderate KF-NAP scores used as the inclusion criteria. However, two of these exhibited deviant rightward bias in one or more of the six VR and eye tracking measures. Conversely, four patients (4, 16, 21, 38) were not picked up by any of the VR and eye tracking measures, but were picked up by different conventional tests. This underlines the heterogeneity of spatial neglect and provides evidence for the complementarity of a VR and eye-tracking based free viewing task for neglect diagnostics. We observed a small rightward gaze bias in the control group's means during 180 s of viewing time, contrary to the leftward bias commonly found in the first 1.5 s by Foulsham et al. (2018). Our measures did not consider the initial gaze behavior.

The free-viewing task in our study resembled findings in group-level midline deviations from previous task-based studies, such as the left/right ratio measured by Kim et al. (2004, 2010) and the head orientation deviation found by Ogourtsova et al. (2018). The very high correlation between head orientation and gaze asymmetry between pictures matches findings from Sidenmark and Gellersen (2019), which showed that focusing on a target further than 15° away typically involves head orientation. We found a median undershooting to the right of the leftmost painting in patients (see Figure 2), similar to what Ogourtsova et al. (2018) observed when their participants approached a left-side (−15°) target in a locomotive task. Since allocentric neglect normally is observed across both sides, we decided not to sub-analyse the within-picture asymmetry on the left side further. Our group based results on fixations mirrored non-VR based studies that found fewer fixations for SN patients on the left side both in free viewing (Fellrath and Ptak, 2015; Ohmatsu et al., 2019) and visual search tasks (Cazzoli et al., 2011). Similarly our gaze asymmetry results and fixations matched the rightward median gaze in SN patients in both free viewing and visual search tasks (Machner et al., 2012, 2018). But in contrast to the study by Primativo et al. (2015) ours did find significant group level differences for fixation durations and counts.

The current free-viewing VR task was not sensitive enough to detect neglect in all patients but there are other behavioral measures that can be derived from the data collected in our setup. For example, Sidenmark and Gellersen (2019) showed that people use different combinations of torso, head, and eye rotations to acquire targets, which might differ for SN patients. Scan paths from eye tracking provide another such avenue that could leverage the spatio-temporal data from viewing each picture (e.g., re-fixations, mean amplitude, saccade landing position, Paladini et al., 2019), and initial fixation location (Foulsham et al., 2018). Our free viewing created no best outcomes but could still be analyzed according to measures similar to Dalmaijer et al. (2015) *quality of search* or *best R*.

Our approach poses some limitations to the results. The simple museum environment and the free-viewing task created low attentional demands. This may have allowed patients to try spending equal amounts of time on each picture if they expected being tested. Tracking of the headset and eye movements may have addressed motor neglect specifically but missing sensory neglect subtypes. The eye tracking data was constrained by a 30 Hz sampling rate and a fairly narrow field of view offered by the current generation head-mounted displays that provided eye tracking. Our results are limited by the accuracy of the gaze tracker both by spatial accuracy and the temporal resolution of the eye tracking data including the inherent jitter. To account for these limitations our measures did not require high spatial accuracy, except for the within-picture measure, which may account for why no significant group difference was found. While neglect patients might perform worse than controls during eye tracking calibration given the spatial arrangement of focal points across the visual field all patients included in our study successfully passed calibration. However, our results indicated opportunities for detailed measurement of behavior over time, rather than "single-instance" tests with potentially less cognitive strain and we saw opportunities for sub-diagnosis on neglect symptoms. Some of the VR and eye tracking measures incorrectly picked up four controls (3, 6, 12, 18). In total 5/96 (5.2%) observations were right-ward bias false positives and 2/96 (2.1%) were left-ward bias false positive, which fairly closely resembles the expected false positive rates from a cut-off criteria set at the 5th percentile. False positives could be reduced by setting a stricter cut-off criteria e.g., at the first percentile. Conventional neglect tests are often confounded by ceiling effects that yield high specificity (true positive rates), while they suffer from low sensitivity (true negative rates) leading to patients passing these tests while still experiencing neglect related problems in more complex everyday activities. In this study, we wanted to compare the sensitivity of VR and eye tracking measures to conventional measure for patients with clinically evident SN measured by the KF-NAP. However, including a group of stroke patients without SN may have revealed subtle gaze biases caused by inter-hemispheric imbalance in this group, too. This may very well be due to the lack of specificity (false negatives) of conventional neglect test, thus VR and eye tracking may in fact increase specificity of SN assessment. This should be investigated in future studies.

Neglect symptoms may be provoked by multitasking, simultaneous stimuli, mental fatigue, stress, or emotional states (Blini et al., 2016). These are intentionally avoided in conventional assessments usually administered in a well-controlled examination room, but could be purposefully exploited in a controlled VR environment. In addition to ceiling effects, many conventional neglect tests lack ecological validity, i.e., providing no direct link between the task tested (e.g., cancellation or line bisection tests) and activities of daily living. This may also be improved in VR and eye tracking based assessment.

This study underlined the heterogeneity of symptoms of SN and represents a first step in using virtual reality in combination with eye tracking measures for individual differential subtype diagnostics from a free viewing context. Virtual reality and eye tracking hold a potential for individual subtype diagnostics that could inform clinical treatment choices and hence treatment efficacy. Improving sensitivity, specificity and ecological validity by use of VR and eye-tracking measures may provide more accurate diagnostics and prognostics for patients with spatial neglect.

## Data Availability Statement

The raw data supporting the conclusions of this article will be made available by the authors, without undue reservation.

## Ethics Statement

The studies involving human participants were reviewed and approved by the Scientific Ethics Committees for the Central Denmark Region. The patients/participants provided their written informed consent to participate in this study.

## Author Contributions

HK, JJ, and LE conceptualized the study and designed the methodology. Analysis of conventional measurements was done by LE and HK. BH and HK were responsible for data verification, cleanup and analysis of VR and eye tracking measures, and visualization of all data. BH created all graphical material and wrote the background with supervision from HK. LE collected and interpreted all clinical measurements. BH, LE, and HK jointly wrote the discussion. All authors contributed to the article and approved the submitted version.

## Conflict of Interest

The authors declare that the research was conducted in the absence of any commercial or financial relationships that could be construed as a potential conflict of interest.

## Publisher's Note

All claims expressed in this article are solely those of the authors and do not necessarily represent those of their affiliated organizations, or those of the publisher, the editors and the reviewers. Any product that may be evaluated in this article, or claim that may be made by its manufacturer, is not guaranteed or endorsed by the publisher.
